# The Effect of Preconditioning Temperature on Gas Permeability of Alkali-Activated Concretes †

**DOI:** 10.3390/ma16114143

**Published:** 2023-06-02

**Authors:** Patrycja Duży, Marta Choinska Colombel, Izabela Hager, Ouali Amiri

**Affiliations:** 1 Chair of Building Materials Engineering, Faculty of Civil Engineering, Cracow University of Technology, 31-155 Cracow, Poland; izabela.hager@pk.edu.pl; 2 Research Institute in Civil and Mechanical Engineering GeM—UMR CNRS 6183, IUT Saint-Nazaire, Nantes University, 44600 Saint-Nazaire, France; marta.choinska@univ-nantes.fr (M.C.C.); ouali.amiri@univ-nantes.fr (O.A.)

**Keywords:** gas permeability, geopolymer concrete, temperature impact, drying

## Abstract

Alkali-activated materials (AAM) are binders that are considered an eco-friendly alternative to conventional binders based on Portland cement. The utilization of industrial wastes such as fly ash (FA) and ground granulated blast furnace slag (GGBFS) instead of cement enables a reduction of the CO_2_ emissions caused by clinker production. Although researchers are highly interested in the use of alkali-activated concrete (AAC) in construction, its application remains very restricted. As many standards for hydraulic concrete’s gas permeability evaluation require a specific drying temperature, we would like to emphasize the sensitivity of AAM to such preconditioning. Therefore, this paper presents the impact of different drying temperatures on gas permeability and pore structure for AAC5, AAC20, and AAC35, which contain alkali-activated (AA) binders made from blends of FA and GGBFS in slag proportions of 5%, 20%, and 35% by the mass of FA, respectively. The preconditioning of samples was performed at 20, 40, 80, and 105 °C, up to the obtainment of constant mass, and then gas permeability was evaluated, as well as porosity and pore size distribution (mercury intrusion porosity (MIP) for 20 and 105 °C). The experimental results demonstrate up to a three-percentage-point rise in the total porosity of low-slag concrete after 105 °C in comparison to 20 °C, as well as a significant increase in gas permeability, reaching up to 30-fold amplification, contingent upon the matrix composition. Notably, the alteration in pore size distribution, influenced by the preconditioning temperature, exhibits a substantial impact. The results highlight an important sensitivity of permeability to thermal preconditioning.

## 1. Introduction

Due to its widespread usage in construction, concrete is subject to a wide range of harsh environmental conditions [1]. There is also a need to reduce CO_2_ emissions due to their harmful influence on climate changes [2,3]. The transportation of external substances within concrete is facilitated by the interconnected pore structure present in the material [4]. For this reason, the characterization of the pore network is of great importance in the assessment of the durability of concrete [5,6].

The gas permeability of a material is a significant parameter in describing its pore structure and is thus considered to be one of the most commonly used indicators for assessing the material’s durability and quality [5,7]. There are several methods used to measure gas permeability, with the Cembureau method and the oxygen permeability index being the most commonly used. These methods involve measuring the quantity of gas that passes through a sample with a known cross-sectional area per unit of time. Typically, gases such as oxygen, nitrogen, or dry air are used in these measurements [8]. It is important to note that these methods are typically conducted in laboratory conditions. While in situ testing methods, such as Figg’s method [9] or others described in the literature [10,11], may be acceptable, their main drawback is the inability to control the impact of temperature and moisture on the material being tested. The strong impact of these parameters on gas permeability cannot be ignored in the analysis of the results [12,13].

There are many characteristics of concrete taken into account in the design process and modelling of the service life of structures; for example, compressive strength, tensile strength, modulus of elasticity, creep, and gas permeability measurements are fundamental in determining the durability of constructions [14]. Although the Cembureau method is commonly used as a reference for measuring air permeability and is considered a benchmark for other measurement methods, it is important to note that laboratory-based methods typically require temperature preconditioning of the specimens [8,15]. The temperature of preconditioning varies from 50 °C to 105 °C. The results of measurements may vary under different conditions. Understanding the impact of temperature on the obtained results can help researchers adjust the testing conditions to suit the materials being tested, enabling meaningful comparisons with other results.

One study by [16] investigated the effect of drying temperature on the gas permeability of AA binders. The authors reported that increasing the drying temperature from 60 °C to 105 °C significantly increased the gas permeability of the material. They attributed this effect to the increase in porosity and microcracking induced by the drying process. However, the study was mainly focused on the water/binder ratio of the binder and its influence on gas permeability.

Another study conducted by Sagbossi et al. [17] showed the impact of elevated temperatures (up to 200 °C) on the gas permeability of hydraulic concretes with different levels of initial saturation. In fact, the changes in geopolymers’ microstructure with temperature have been the subject of few investigations [18,19]. Some of these have included investigations into high temperatures of up to 1000 °C [20,21]. As has been established, the temperature at which curing takes place has a significant impact on the properties of geopolymer materials, particularly those that involve fly ashes [22,23]. The microstructure and porosity of AA slag are also affected by the temperature of curing [24]. Considering the influence of specimen preconditioning on gas permeability, the applicability of such methods to geopolymer concretes remains an unresolved issue.

For many years, researchers have investigated the temperature that activates fly ashes [25,26]. Palomo et al. [27] described the evolution of the properties of AA fly ash exposed to temperatures between 65 °C and 85 °C. High temperature exposure enabled them to reach an increase of compressive strength of up to 60 MPa. The impact of alterations in the curing temperature on several properties associated with porosity is evident. These studies have provided further confirmation of theories proposed much earlier [28]. Similar studies have been conducted to determine the impact of activators used [29] for mixes and their properties in temperatures between 75 °C and 95 °C. Unfortunately, there is no universal rule that can describe the impact of curing temperature on the porosity and microstructure of AA materials. The outcome depends on the types of precursors and activators, as well as the temperature applied. In terms of gas permeability measurement, it is more consistentto compare the results obtained for materials with different compositions than to obtain precise values of permeability. This paper presents the influence of preconditioning temperature on gas permeability measurements and compares this impact across various compositions of AACs.

## 2. Materials

The studies were conducted on six compositions of AACs based on fly ash (Połaniec powerplant, Poland) replaced by mass with ground granulated blast furnace slag (Ekocem Dąbrowa Górnicza, Poland). The influence of different dosages of ground granulated blast furnace slag (GGBFS) was examined at three levels: 5%, 20%, and 35%. In the presented research, three precursors can be distinguished, each comprising two components in varying proportions. Two types of coarse aggregates, basalt and dolomite, were used to investigate their impact on gas permeability. The tested blends were labeled as AAC5B, AAC20B, and AAC35B for basalt aggregate, and AAC5D, AAC20D, and AAC35D for dolomite aggregate, with 5%, 20%, and 35% of slag, respectively. The fly ash (FA) used in this study was classified as class F according to ASTM C618 Standard Specification for Coal Fly Ash and Raw or Calcined Natural Pozzolan for Use in Concrete [30], and it is primarily composed of silicon dioxide and aluminum oxide. The slag used in the study was mainly composed of calcium oxide and silicon oxide. The chemical compositions of the precursors’ components are presented in Table 1.

**Figure 1:**
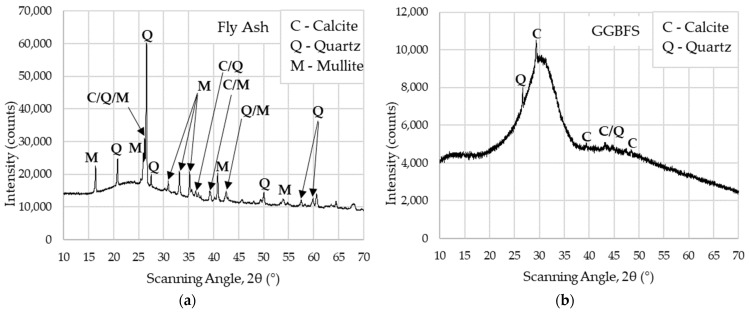
XRD analysis of precursor’s composition: (**a**) FA, (**b**) GGBFS.

The degree of crystallinity of the precursors has a significant impact on the chemical activation and hardening of the binders in AAMs [31]. Figure 1a,b depict the X-ray diffraction (XRD) results, which offer an overall understanding of the phases of the precursors used in this study.

Numerous crystal structures were identified during the fly ash analysis, which is consistent with other findings reported in the literature [29,32]; mainly mullite, quartz, and calcite were detected. In the case of GGBFS, only a few crystalline phases were detected, including calcite and quartz. All discernible peaks shown in the graphs correspond to the FA and GGBFS crystalline phases. Amorphous phases, represented by the rest of the diagram, show a significant tendency towards fast chemical reactions. This feature contributes to the acceleration of the setting time of the concrete mix. For activation, a diluted sodium silicate solution Geosil^®^ 34417, supplied by Woellner, was used. Specifications declared by the manufacturer are listed in Table 2.

The mixing procedure was carried out based on the previous experience of the research team [33]. The initial step involved the preparation of pastes. A homogeneous mixture was achieved by blending the fly ash with a diluted alkaline solution. Subsequently, the slag was added to the mixture while constantly stirring to avoid the formation of clumps. Subsequently, a blend of coarse aggregate (either basalt or dolomite) and quartz sand was mixed in a larger mixer. Once the paste was prepared, it was added to the mixer containing the aggregate and mixed uniformly. The resulting mixture was then poured into cylindrical moulds, 11 cm in diameter and 22 cm in height, and compacted using a shaking table. They were stored in ambient conditions with a temperature of 20 ± 2 °C and protected from water evaporation by plastic film. The samples that were prepared did not undergo thermal curing and were removed from the moulds after one day, except for the concrete containing 5% of slag, which was taken out after two days.

## 3. Methods

**Figure 2:**
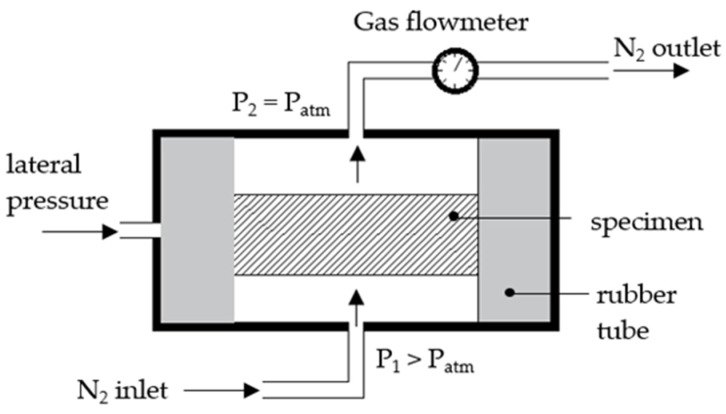
Cembureau setup for gas permeability measurement of concrete.

The Cembureau method remains a widely used technique for measuring gas permeability [34]. The pressure values of the inlet (P1) and outlet (P2) are kept constant during the measurement to obtain accurate results for gas permeability. The measurement is based on a stabilized and permanent gas flow. The measurement also accounts for the atmospheric pressure and temperature, which can potentially impact the results, to accurately estimate the permeability. Standard inlet pressure values are between 1 bar (0.1 MPa) and 5 bar (0.5 MPa). The rubber gum surrounding the specimen is subjected to a lateral pressure of 8 bars to prevent gas from leaking around the sample. Figure 2 illustrates the setup of the Cembureau method.

**Figure 3:**
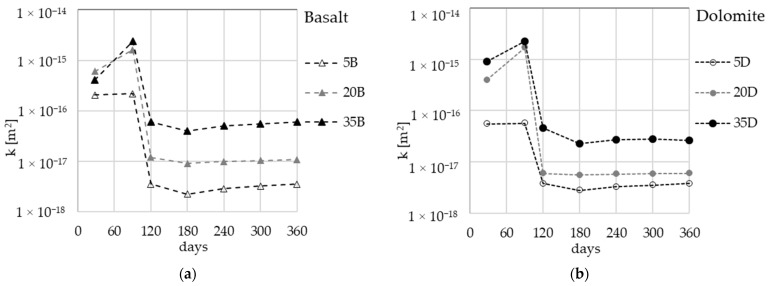
Gas permeability development over time for samples stored in room conditions: (**a**) basalt aggregate, (**b**) dolomite aggregate.

Gas permeability measurements were conducted following the standard method, using samples that had not been previously dried, being stored in room conditions. The stability of the gas permeability value was observed to begin after 240 days, and the measurements were continued up to 360 days, as shown in Figure 3.

To prevent any interference in the test results due to the age of materials, the effect of pre-conditioning temperature was studied after the permeability values had stabilized (after 1 year). As AAMs are known to be extremely sensitive to changes with temperature, four pre-conditioning temperatures (20 °C, 40 °C, 80 °C, and 105 °C.) were selected for this study.

**Figure 4:**
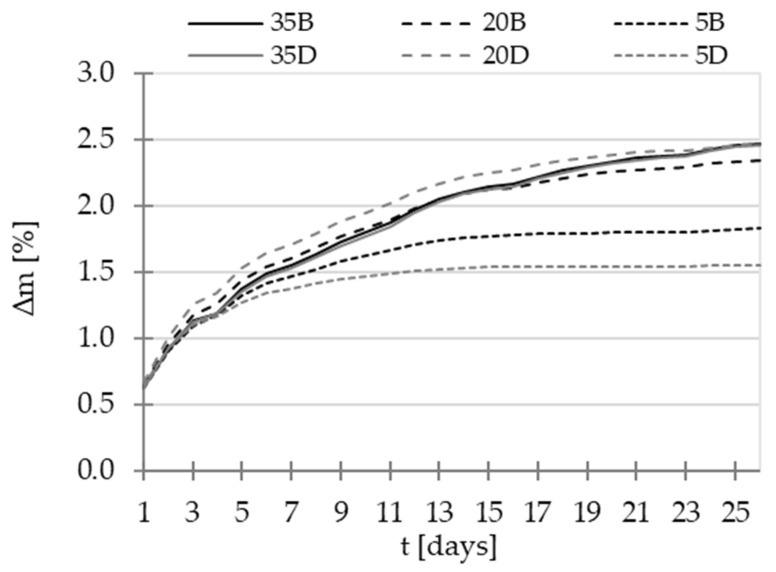
Mass stabilization in the drying process at 40 °C.

Following the first measurement step conducted at the natural state of the material (referred to as 20 °C, based on the data at 360 days, as presented in Figure 3), subsequent stages of the study were carried out. These stages involved subjecting the samples to drying processes at 40 °C, 80 °C, and finally 105 °C. For each step of temperature, the drying process was carried out to reach a stable mass of samples (criteria of relative mass loss ∆m inferior to 0.1% during 24 h). The example of mass stabilization for drying at 40 °C is presented in Figure 4.

**Figure 5:**
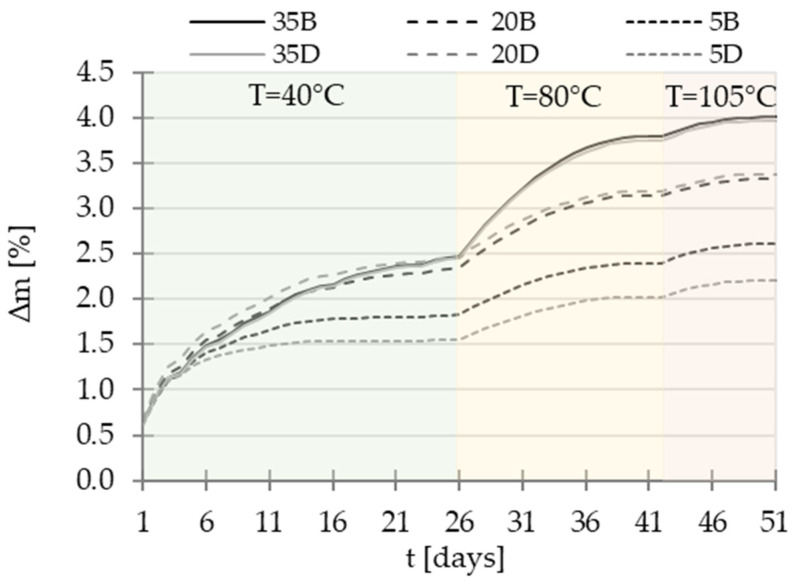
The graph of the drying process.

To cool down the specimens, they were placed in a desiccator filled with hydrophobic pellets to minimize moisture presence. After 24 h of cooling, the mass of each material was checked, and then the permeability test was conducted. Immediately after the measurements, the specimens were placed in a dryer with the next level of temperature, and the procedure was repeated. All tests were performed on the same specimens to minimize the impact of heterogeneity of concretes. The complete drying process and the changes in the mass of the specimens are depicted in Figure 5.

The values of gas permeability coefficients have been calculated according to modified Darcy’s law (Equation (1)):

(1) $$k_{A}=2µL\frac{P_{1}}{P_{1}^{2}-P_{2}^{2}}\frac{Q}{A}$$

where the abbreviations and measurements are as follows:

- *k_A_*—apparent permeability, m^2^;
- µ—viscosity of the gas at 20 ± 2 °C, Pa × s;
- *L*—thickness of the specimen, m;
- *P*_1_—inlet pressure, Pa;
- *P*_2_—outlet (atmospheric) pressure, Pa;
- *Q*—flow rate, m^3^/s;
- *A*—cross-sectional area of the specimen, m^2^.

For nitrogen, the value of viscosity µ at the temperature of 21 °C is equal to 1.77 × 10^−5^ Pa × s [35]. The analysis of the results obtained focuses on the impact of temperature on both the apparent and intrinsic gas permeability values, as well as the presence of the boundary slippage effect that is associated with the Klinkenberg effect [36].

The analysis of microstructure (porosity and pore size distribution) was evaluated with the use of MIP for the specimens treated at 20 °C and 105 °C. The measurement involves the application of increasing pressure to a sample immersed in mercury, causing the mercury to penetrate the material’s pores. As the pressure increases, the size of the pores that mercury can penetrate becomes smaller, and the intrusion volume is measured at each pressure increment. The measurements were performed using a Poromaster Micromeritics AutoPore IV. For each type of concrete (six types at two temperatures), at least three samples of size approx. 2 cm^3^ were cut from the cylinder samples. The average values of total porosity, tortuosity, and pore size distribution for each material for 20 and 105 °C highlight the impact of preconditioning temperature on the microstructure of AA concretes.

## 4. Results and Discussion

**Figure 6:**
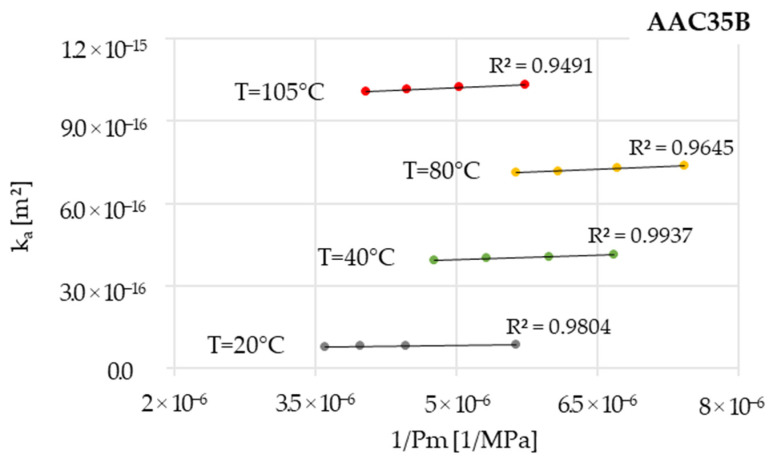
The results of gas permeability measurements for AAC35B.

Apparent permeabilities in the function of inversed mean pressure show that permeability increases with temperature and follows Klinkenberg’s law (see Figure 6 for AAC35B as an example). The slopes’ increase with higher temperatures means that pore fineness is increasing, probably because of the creation of newly accessible fine pores during drying and cooling. Simultaneously, an opposing effect can occur due to the expansion of existing pores that are already accessible, which is caused by the evaporation of water from their internal surfaces [37,38].

The temperature effect shown in Figure 6 can be clearly explained using mercury porosimetry analysis. The total mercury porosity of the tested materials at the temperatures of 20 °C and 105 °C is presented in Table 3. One can observe that total porosity decreases with slag content for any type of aggregate used, as well as for any pre-treatment temperature. However, the pre-treatment temperature causes total porosity to increase for almost all of the materials. The experimental results demonstrate an up to three-percentage-point rise in the total porosity of low-slag basalt concrete (AAC5B) after 105 °C in comparison to 20 °C.

**Figure 7:**
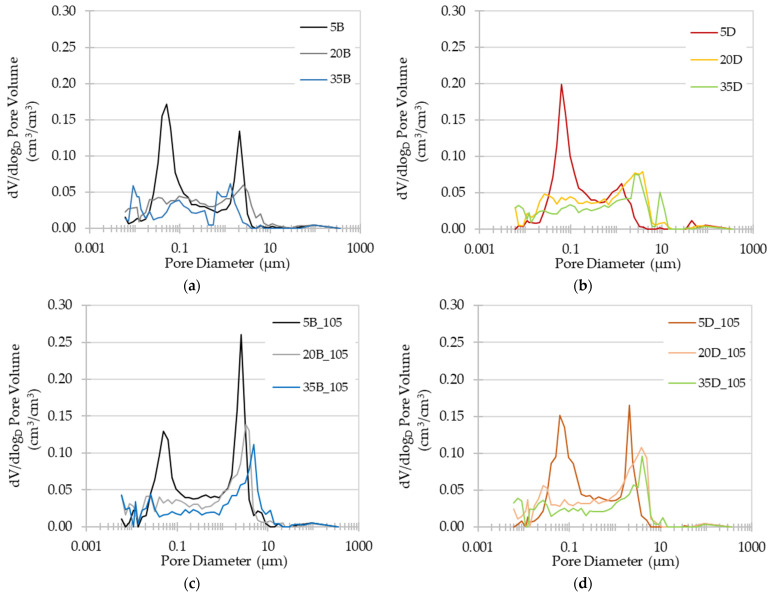
Pore diameter distribution of tested materials: (**a**) basalt concretes at a temperature of 20 °C; (**b**) dolomite concretes at a temperature of 20 °C; (**c**) basalt concretes at a temperature of 105 °C; (**d**) dolomite concretes at a temperature of 105 °C.

In the case of the gas permeability of concrete, it is more dependent on the diameter of the pores present in the material. The influence of temperature on the change in the pore diameter distribution in the tested concretes is shown in Figure 7a–d.

The presented graphical data provide an illustration of the influence of temperature on the pore size distribution within the tested materials. We can observe two types of impact of thermal treatment on pore size distribution. In the case of low-slag concretes, the dominant pore size diameter after pre-treatment at 20 °C moves towards larger pores after pre-treatment at 105 °C, while still maintaining a tendency for one pore size to dominate in a specific material. The dominant pore diameter after preconditioning at 20 °C was 0.05 µm, and 2.09 µm for AAC5B and 0.06 µm for AAC5D (see Figure 7a,b). After pre-treatment at 105 °C, the dominant pore diameter increased to 2.55 µm for AAC5B, and it increased to 2.08 µm from 0.06 µm for AAC5D. The second tendency can be observed for concretes with 20% and 35% slag content. For these materials, after pre-treatment at 20 °C, the pore size distribution did not indicate the dominant pore diameters of analysed materials. After heating at 105 °C, the appearance of pores that quantitatively exceeded the others was noted. The diameters of the most common pores in these concretes were 3.22 µm, 4.86 µm, 3.85 µm, and 3.89 µm for AAC20B, AAC35B, AAC20D, and AAC35D, respectively. After pre-conditioning the materials at 105 °C, the materials were finally dominated by larger pores than after pre-conditioning at 20 °C, which had a strong effect on the tested gas permeability, as it is discussed below.

Table 4 presents intrinsic permeability values for not oven-dried, stored in room conditions (20 °C) specimens of all the tested concretes. These intrinsic permeabilities were calculated following Klinkenberg’s law, using apparent permeabilities, as shown before in Figure 6 for one exemplary material. The materials with low slag content (AAC5) have the lowest values of permeability, regardless the type of aggregate used, while there is no clear trend in permeability evolution as a function of aggregate type for all the materials tested.

**Figure 8:**
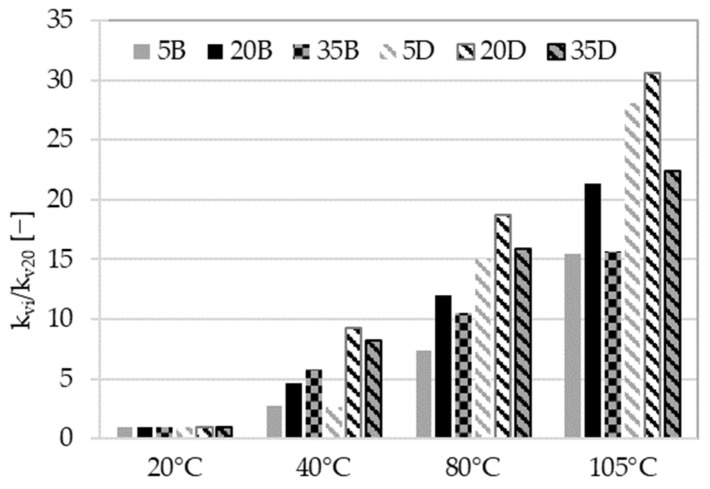
Values of k_i_/k_20_ ratios for different temperatures of preconditioning.

The following analysis is focused on the intrinsic gas permeability values and their correlation with specific drying temperatures. The ratio proposed for further analysis is k_i_/k_y_, where i and y refer to drying temperatures. Values of k_i_/k_20_ are shown in Figure 8.

The permeability values for concrete with dolomite aggregate increased more significantly than for those with basalt aggregate for almost all of the tested temperatures (40 °C, 80 °C, and 105 °C). This may be due to physical changes in the microstructure of the zone between the paste and the aggregate, as the latter are not intrinsically affected at these temperatures. Figure 8 clearly shows that the drying process at 40 °C had a greater impact on the intrinsic gas permeability values of AAC35B and AAC35D compared to AAC5B and AAC5D. For AAC35B, the value of permeability increased from 6.07 × 10^−17^ m^2^ to 34.8 × 10^−17^ m^2^ between 20 °C and 40 °C, respectively (k_40_/k_20_ ratio equal to 5.74). For AAC35D, permeability for these temperatures reached 4.65 × 10^−17^ m^2^ and 38.2 × 10^−17^ m^2^, which makes a k_40_/k_20_ ratio equal to 8.22. However, low-slag-content materials showed a lower sensitivity when heated to the temperature of 40 °C. The value of the k_40_/k_20_ ratio for AAC5B and AAC5D were 2.71 and 2.64, respectively.

**Figure 9:**
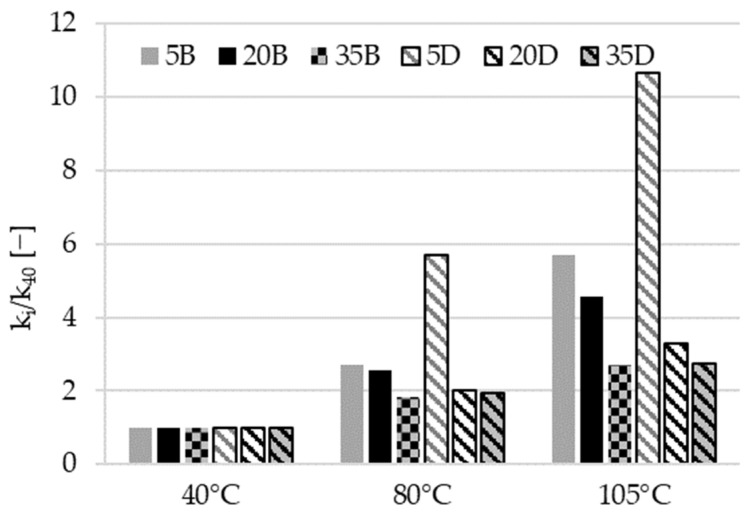
Values of the ratio k_i_/k_40_ ratios for temperatures of preconditioning exceeding 40 °C.

The impact of the drying process over 40 °C is related to complex physical and chemical changes and will be discussed below. Values of the ratio k_i_/k_40_ are shown in Figure 9.

Materials with a high fly ash content showed a notable reaction to temperatures ranging from 40 °C to 105 °C. As discussed earlier, these materials are susceptible to thermal treatment. This susceptibility may be due to the activation of the fly ash and consequent formation of new phases, as well as to the quick removal of water from the material’s pores (as presented in Figure 7c,d) and the generation of microcracks, probably at the interface paste-aggregate, which enhance gas flow.

**Figure 10:**
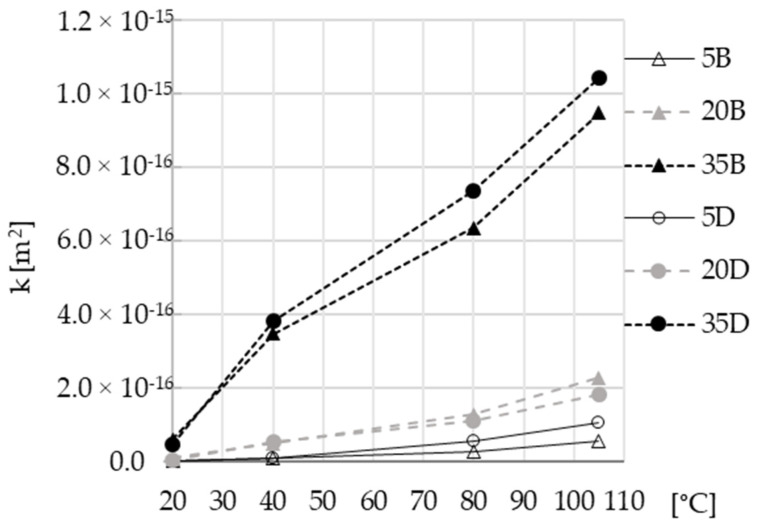
Impact of drying temperature on intrinsic gas permeability of AAC.

All of the absolute values of intrinsic gas permeability are summarized in Figure 10.

**Figure 11:**
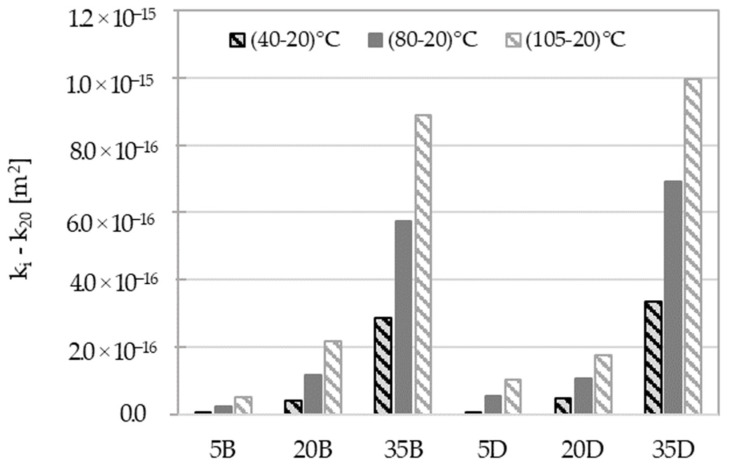
Differences of intrinsic gas permeability as the subtraction of their values.

The previous analysis of the gas permeability of concretes was mainly related to the nature of the aggregate. However, the results presented in Figure 10 primarily focus on the impact of slag content. Three groups of results can be distinguished, depending on the amount of slag. For materials with 5% and 20% slag content, the influence of the drying temperature is slight compared to those with 35% slag. The difference between values of permeability for different temperatures and reference at 20 °C are displayed in Figure 11. The observed MIP changes in the microstructure of materials (see Figure 7), particularly the appearance of a dominant pore diameter for higher-slag-content concretes, may explain the permeability increases depicted in Figure 11. Moreover, the thermal expansion strain discrepancies between the paste with high slag content and the aggregate should be more significant than for other pastes. This leads to geometric incompatibilities, which may cause microcracking, resulting in an increase in permeability by up to two orders of magnitude, as shown in Figure 11.

The analysis that was conducted has provided numerous valuable observations. Figure 11 illustrates the differences in the impact of drying temperature and shows that the materials are sensitive to drying in different ways. In addition, the significant impact of slag content is emphasized.

## 5. Conclusions

The objective of this study was to examine the effect of the preconditioning temperature of the AAC specimens on the outcome of the gas permeability test. The use of different measurement standards can effectively have a significant impact on the results, as demonstrated in this experimental study. The following conclusions can be drawn:

- The obtained permeability values are significantly affected by the drying temperature of the samples: the permeability may attain over a thirty-fold increase of its initial value after pretreatment at 105 °C;
- The effect of temperature on AAC permeability is strongly related to the precursor used and the nature of the aggregate. In general, the materials with low slag content (AAC5) have the lowest values of permeability for all the types of aggregate used. However, the permeability values for AAC with dolomite aggregate increase more significantly than for those with basalt aggregate for almost all of the tested temperatures;
- The main causes of permeability increases with temperature preconditioning are microstructure changes linked particularly with pore size increases, while the total porosity impact seems to be negligible;
- The interplay between preconditioning temperature and pore structure in concretes is a complex phenomenon that is contingent upon the composition of the precursor and the coarse aggregate used. These two factors independently modulate the gas permeability of concrete under varying temperature conditions;
- Permeability test reports need to include sample preconditioning conditions to enable the correct interpretation of results;
- The gas permeability of materials with the same binder could be compared under the same standardized conditions, taking into consideration the influence of aggregate type and the quality of the zone between the paste and the aggregate.

This experimental research has shed light on the feasibility of comparing results obtained from materials with varying binders and aggregates. The conclusions drawn from these studies can serve as guidance for further analysis and interpretation of permeability test results conducted on AAC.

## Figures and Tables

**Table 1:** Oxide composition of fly ash and ground granulated blast furnace slag.

wt.%	SiO_2_	Al_2_O_3_	Fe_x_O_y_	CaO	MgO	SO_3_	K_2_O	Na_2_O	P_2_O_5_	TiO_2_	Mn_3_O_4_	Cl^−^
FA	52.30	28.05	6.32	3.05	1.71	0.28	2.51	0.76	0.69	1.35	0.07	-
GGBFS	39.31	7.61	1.49	43.90	4.15	0.51	0.36	0.47	-	-	-	0.04

**Table 2:** Chemical composition of alkaline solution Geosil^®^ 34417.

Characteristic	Unit	Woellner Geosil^®^ 34417
Na_2_O content	wt.%	16.74
SiO_2_ content	wt.%	27.5
Density	g/cm^3^	1.552
Viscosity	mPa × s	470
Weight ratio (WR = wt.% SiO_2_/wt. Na_2_O)	-	1.64
Molar ratio (MR = mol SiO_2_/mol Na_2_O)	-	1.70

**Table 3:** The total mercury porosity of tested materials at the temperatures of 20 °C and 105 °C.

	AAC5B	AAC20B	AAC35B	AAC5D	AAC20D	AAC35D
Porosity 20 °C (%)	14.87	11.77	7.30	14.51	13.27	10.91
Porosity 105 °C (%)	17.96	13.14	10.44	15.42	13.57	9.94

**Table 4:** The intrinsic permeability values for specimens preconditioned at 20 °C.

	AAC5B	AAC20B	AAC35B	AAC5D	AAC20D	AAC35D
k_20_ (m^2^)	3.53 × 10^−18^	1.08 × 10^−17^	6.07 × 10^−17^	3.81 × 10^−18^	6 × 10^−18^	4.65 × 10^−17^

## Data Availability

The data presented in this study are available on request from the corresponding author.
